# Antimicrobial and Antibiofilm Coating of Dental Implants—Past and New Perspectives

**DOI:** 10.3390/antibiotics11020235

**Published:** 2022-02-11

**Authors:** Guilherme Melo Esteves, João Esteves, Marta Resende, Luzia Mendes, Andreia S. Azevedo

**Affiliations:** 1 FMDUP—Faculty of Dental Medicine, University of Porto, Rua Dr. Manuel Pereira da Silva, 4200-393 Porto, Portugal; guiesteves11@gmail.com (G.M.E.); up201404016@edu.fmd.up.pt (J.E.); martasantosresende@gmail.com (M.R.); lgoncalves@fmd.up.pt (L.M.); 2 LEPABE—Laboratory for Process Engineering, Environment, Biotechnology and Energy, Faculty of Engineering, University of Porto, Rua Dr. Roberto Frias, 4200-465 Porto, Portugal; 3 i3S—Instituto de Investigação e Inovação em Saúde, Universidade do Porto, Rua Alfredo Allen 208, 4200-135 Porto, Portugal; 4 IPATIMUP—Institute of Molecular Pathology and Immunology, University of Porto, Rua Júlio Amaral de Carvalho 45, 4200-135 Porto, Portugal

**Keywords:** oral biofilm, dental implants, titanium implants, antimicrobial, surface coating, anti-fouling

## Abstract

Regarded as one of the best solutions to replace missing teeth in the oral cavity, dental implants have been the focus of plenty of studies and research in the past few years. Antimicrobial coatings are a promising solution to control and prevent bacterial infections that compromise the success of dental implants. In the last few years, new materials that prevent biofilm adhesion to the surface of titanium implants have been reported, ranging from improved methods to already established coating surfaces. The purpose of this review is to present the developed antimicrobial and antibiofilm coatings that may have the potential to reduce bacterial infections and improve the success rate of titanium dental implants. All referred coating surfaces showed high antimicrobial properties with effectiveness in biofilm control, while maintaining implant biocompatibility. We expect that by combining the use of oligonucleotide probes as a covering material with novel peri-implant adjuvant therapies, we will be able to avoid the downsides of other covering materials (such as antibiotic resistance), prevent bacterial infections, and raise the success rate of dental implants. The existing knowledge on the optimal coating material for dental implants is limited, and further research is needed before more definitive conclusions can be drawn.

## 1. Introduction

Replacing missing teeth with dental implants is one of the most common treatment options with a great success rate. However, they still fail a significant number of times due to infections such as peri-implant mucositis, a biofilm-induced inflammation that can trigger bone loss, and result in peri-implantitis [1,2,3,4,5,6,7,8].

The success of oral rehabilitation using dental implants depends on numerous factors. The implantation process requires good interactions between the titanium surface and surrounding bone tissue (osseointegration), as well as resistance against bacterial colonization since implant-related infections are responsible for a large part of implant failure [9].

**Figure 1:**
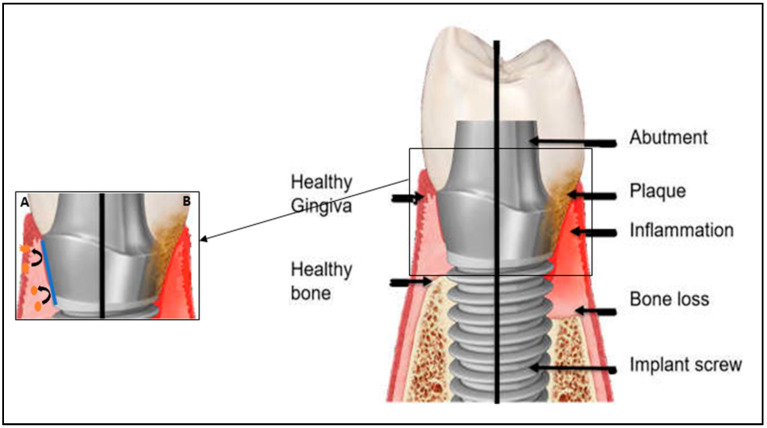
Biofilm formation process around dental implants and dental structures. (**A**) Implant coating prevents microbial colonization. (**B**) Dental plaque formation.

Studies indicate that around 29.48% (implant-based) and 46.83% (subject-based) of dental implants suffer from peri-implant mucositis and around 9.25% (implant-based) and 19.83% (subject-based) develop peri-implantitis [10]. Peri-implant mucositis is a biofilm-induced inflammation localized on the soft peri-implant mucosa, without any evidence of supporting bone loss (Figure 1B) [8]. It develops from healthy peri-implant mucosa around osseointegrated dental implants after the accumulation of bacterial biofilms. The major clinical sign of peri-implant mucositis is bleeding on probing (BOP), although it can also present erythema, swelling, and suppuration [2]. Clinical studies reported reversibility of peri-implant mucositis state after at least three weeks of better oral hygiene and biofilm control [3]. Nonetheless, if left untreated, the inflammatory process may progress and trigger the gradual destruction of the bone surrounding the implant, resulting in peri-implantitis [8].

These types of infections, when untreated, result in implant loosening and require implant removal [4]. The ideal dental implant should have both great osseointegration properties and protection from the bacteria that cause peri-implant mucositis [2,3]. To achieve that, titanium and its alloys were chosen as the go-to material in commercialized implants due to its properties such as great resistance to corrosion, biocompatibility, and good tolerance by the biological environment, amongst others [2,3,5,7].

In order to improve success rates, modifications to the implant surface were proposed and studied. Changing the surface’s physic and chemical characteristics such as roughness, surface free energy, and wettability allowed improving osseointegration [5]. The next step was to control and prevent the accumulation of bacteria around the implant since bacterial adhesion occurs immediately after implantation and results in biofilm formation. Biofilm is also resistant to many antimicrobial agents, making it difficult to treat once established [11]. In order to prevent bacterial infections, surface coatings with antimicrobial properties were hypothesized to be a reliable solution for this problem.

Titanium implants are susceptible to bacterial adhesion (Figure 1B) depending on the implant surface [12]. In order to prevent bacterial colonization, the titanium surface may be treated by adding materials or agents in the form of coatings (Figure 1A) [11].

Coating materials such as silver, copper, zinc, chlorhexidine, and some antibiotics presented to be a promising solution due to antimicrobial properties that would fight bacterial colonization [4,9]. Nonetheless, the methods required to modify and incorporate coatings in the implant surface are complex and expensive. Furthermore, while trying to achieve maximum antimicrobial properties, biocompatibility and osseointegration properties may be lost. The balance will always be key to determining the potential of a coating [13]. An ideal implant should have both osseointegration and antimicrobial properties [4,14]. There are numerous coatings developed throughout the years with promising results enhancing antimicrobial properties, achieved by either physical or chemical modification or even a combination of both, and we will discuss the most relevant ones.

This review aims to examine a wide range of coating materials and procedures in order to determine which novel solutions offer the best chances of producing a viable anti-fouling surface coating for dental implants.

## 2. Materials and Methods

### 2.1. Search Strategy and Information Sources

Four electronic sources of evidence were consulted in search of suitable articles that matched the aim of this review: MEDLINE (via PubMed), Scopus (Elsevier: Amsterdam, The Netherlands), Web of Science (Clarivate Analytics: Philadelphia, PA, United States), and SciELO (Scientific Electronic Library Online: Brazil), up to 28 May 2021.

Papers were searched using the following keywords: “Dental implant”; “Periodont*”; “Periodontal pockets”; “Microbes”; “Oral biofilm”; “Micro *”; “Oral micro *”; “Bacteria”; “Surface coating”; “Antimicrobial”; “Antibacterial”; “Titanium implants”; “Antibiotic loaded coating; “Biosurfactant”; “Chlorhexidine coating”; “Polymer coating”; “Implant infections”; “ Anti-infective biomaterials”; “Antiadhesive surfaces”; “Nanostructured materials”; “Antibiofilm molecules”; “Antisense peptide nucleic acids”; “Drug delivery”; and “Antimicrobial photodynamic therapy”. The keywords were combined with Boolean operators “AND” or “OR” with proximity operators [“” and ()] and with the truncation operator (*) used whenever appropriate.

The electronic database search was supplemented with a hand search across the references of all included papers.

### 2.2. Eligibility Criteria

The search was designed to be as broad as possible, with the goal of including all studies that alluded to coatings treatments on titanium surfaces to test their applicability on dental implants. All studies with less than ten years of publishing data were included. Restrictions were made to article type by excluding reviews, thesis, case reports, or letters.

## 3. Results

The most notable coatings, as well as their most important qualities and properties, are described in this section.

### 3.1. Bacteriostatic Materials

Various molecules show bacteriostatic properties, which means that they can repel bacteria from the surface of the implant without killing it [9].

Polymers such as polycations and biosurfactants have been studied and applied to titanium surfaces, being able to provide bacteriostatic properties to titanium surfaces. Moreover, recent studies were able to combine bactericidal and bacteriostatic materials granting both properties to titanium surfaces [9].

#### 3.1.1. Polymer Coatings

Polyethylene glycol (PEG) is one of the most widely used polymers that provide antifouling properties to material surfaces, namely titanium surfaces [15,16]. It has excellent bacteriostatic qualities due to its hydrophilic and flexible chains. However, the very efficient antibacterial repelling properties also inhibit eukaryotic cell attachment (e.g., osteoblasts), thus compromising osseointegration. Therefore, they require the addition of cell adhesive sequences such as RGD (arginine-glycine-aspartate) peptides to preserve their biocompatibility [15].

Aiming to prevent the adhesion of bacteria to the surface of medical implants, polymer coatings with hydrophobic polycations such as N,N-dodecyl,methyl-PEI, as described by Schaer et al. [17], were studied and not only have shown a significant reduction in bacterial colonies of *S. aureus* when coated in titanium surfaces in vitro but also when applied in sheep models in vivo. Membrane proteins, teichoic acids (Gram-positive bacteria), and negatively charges phospholipids (Gram-negative bacteria) grant a negative surface charge to microbial cells. Polycations are attracted to the negativity present in microbial cells’ surface, and based on their amphiphilic properties, they can disrupt their membrane and enable cell lysis, and this results cell death, adding bactericidal potential to the polymer coating.

Unfortunately, fabrication of the coating structure is a costly and challenging process, and there is a risk of polymer degradation with time, which could compromise the long-term stability and effect of the coating surface. Moreover, some polymer coatings are not yet available for use in titanium dental implants since the process of screwing the implants to the bone would compromise the structure of the coating, making it an unviable option [9]. Nonetheless, when a stable structure of polymers and cell adhesive sequences is achieved, both anti-biofouling and osseointegration results are expected [15].

#### 3.1.2. Totarol

Totarol is a natural antibacterial agent that presents to be a promising solution towards the prevention of biofilm formation [14]. Clinically efficient against *Methicillin-resistant Staphylococcus aureus* and with demonstrated low cytotoxicity, Totarol was hypothesized and tested as a coating surface for titanium implants [14].

Xu et al. [14] analyzed the behavior of Totarol coated titanium disks with *Streptococcus gordonii* and human saliva. After 24 h, all bacteria were killed when compared to the control group, demonstrating the bactericidal effect of Totarol against *S. gordonii*. When tested for the long-term antibacterial effect, it was noted that the bactericidal effect was weakened after 12 days, but bacteriostatic mechanisms, namely anti-adhesion and anti-aggregation, were still inhabiting *S. gordonii* proliferation on the titanium surface, even after 24 days, while maintaining biocompatibility.

Improvements can be made to this surface coating, mainly to the long-lasting efficiency of antibacterial properties. Nonetheless, Totarol is another promising candidate to prevent peri-implantitis in the healing stage of the implantation process [14].

#### 3.1.3. Biosurfactants

Biosurfactants are the most recent addition to the list of possible coatings with antibacterial properties for dental implants. Tambone et al. [18] conducted the only study using rhamnolipids on a titanium surface.

Rhamnolipids are a microbial surfactant mainly produced by *Pseudomonas aeruginosa.* They can preserve the biocompatibility of the titanium surface due to their low cytotoxicity and restrain the microbial adhesion process due to their amphiphilic structure. They can also modify permanently cell membranes, which could result in cell lysis [18].

The coated titanium disk with 4 mg/mL of rhamnolipid solution was tested against *Staphylococcus aureus* and *Staphylococcus epidermidis* for 72 h. After 24 h, *S. aureus* inhibition was higher than 90%, and *S. epidermidis* inhibition ranged from 62 to 78% depending on titanium surface morphology. After 72 h, the reduction in *S. aureus* was about 7% and 10.3% for *S. epidermidis*. No cytotoxicity was verified on any coated surface [18].

Rhamnolipids seemed to be another promising strategy for reducing both bacterial adhesion and biofilm reduction on titanium surfaces.

### 3.2. Bactericidal Materials

Some of the known strategies to lower bacterial load include damage to the bacteria’s membrane or cell wall, penetration of the cell wall, DNA damage that hinders bacteria multiplication, creation of reactive oxygen species (ROS), blocking of ATP synthase, and stopping cell respiration [9,19]. Some materials imbued in surface coatings can grant bactericidal properties to titanium dental implants through some of the mechanisms mentioned and prevent biofilm formation.

#### 3.2.1. Antimicrobial Peptides (AMP)

Antimicrobial peptides are a potential solution against biofilm colonization on titanium dental implants due to their antimicrobial properties. Geng et al. [20] studied engineered chimeric peptides with antimicrobial activity and concluded that, despite the need for further studies, these peptides had promising results regarding antimicrobial activity. Zhou et al. [21] studied a cationic antimicrobial peptide, GL13K, and by using X-ray photoelectron spectroscopy and ultrasonication showed that this AMP improved both antibacterial and cytocompatibility properties of titanium implants, greatly inhibiting biofilm growth in vitro of *P. gingivalis* cultures, in the first 12 h, when compared to the control group. After 72 h, the antibacterial effect of GL13K coated surfaces was less effective but still an improvement when compared to uncoated titanium surfaces.

AMPs can be a good alternative to commonly used antibacterial materials such as silver, due to their flexibility, since they possess both antibacterial and osseointegration properties. Despite having a broad spectrum of action against bacteria, they appear to have a lower propensity to develop antibacterial resistance and toxicity [15,16].

Despite promising results, bioactive coatings with AMPs require complex designs of synthetic peptides that are quite costly to fabricate, which may compromise their broad use in titanium dental implants [9].

#### 3.2.2. Ion-Implanted Surfaces

Ions from elements such as fluorine (F), copper (Cu), zinc (Zn), chlorine (Cl), iodine (I), selenium (Se), or cerium (Ce) can be incorporated into coatings in titanium implants [9]. Additionally, Bismuth (Bi) has recently been proposed as an antibacterial addition for calcium phosphate cement and titanium surfaces [22]. Zhou et al. [23] evaluated the potential of doped fluorine in Ti02/calcium-phosphate coatings (TiCP). With three different amounts of fluorine in the coating designated TiCP-F1 (least amount of fluorine), TiCP-F6, and TiCP-F9 (most amount of fluorine), they concluded that the TiCP-F1 coating had higher osteogenic properties than pristine (uncoated) titanium, but lacked antibacterial properties. On the other hand, TiCP-F6 and TiCP-F9 coatings had increased amounts of fluorine and showed significantly improved osteogenic and antibacterial properties.

One common coating applied to titanium surfaces is calcium-phosphate (CaP) due to its bioactive and osteoconductive properties [4]. Aranya et al. [4] modified CaP’s surface by doping it with fluoride and zinc ions, both alone and combined. Fluoride is known for its bactericidal effect while Zinc is more associated with osseointegration promotion, despite also showing antibacterial properties [4].

They studied the effectiveness of this coating against *P. gingivalis* [24,25]. FZn-CaP coating had great results regarding inhibition of bacterial adhesion with ~88% reduction when compared to uncoated control disks in the first 72 h. F-Cap and Z-Cap coatings each had ~89% reduction in bacterial adhesion. After 7 days, biofilm reduction was significantly lower for both coatings. Zinc and Fluoride doped into CaP coating is a great option for dental implants since it enhances titanium surfaces with both bactericidal and bioactive properties [4].

Shen et al. [26] studied and verified that incorporating Zn ions in titanium dental implants surface coatings reduced the growth of *P. gingivalis*. Lin et al. [27] used Bismuth (Bi) to chemically modify titanium implants and was able to reduce *S. mutans* colonization.

A variety of tested ions also proved to be a promising solution to grant antimicrobial properties to the surface of titanium dental implants although they still lack long-term effects.

#### 3.2.3. Photoactivatable Bioactive Titanium

Titania or titanium dioxide (TiO_2_) is a nanocomposite coating with antimicrobial properties once it is photo-activated [9]. Under strong UV light, reactive oxygen species (ROS) are generated, which allows TiO_2_ to kill a wide range of microorganisms such as bacteria while maintaining biocompatibility [9,28].

TiO_2_ properties such as its’ low-cost, stability, reactivity, durability, biocompatibility, and corrosion resistance make it a great option for commercial antimicrobial coatings. Thus, it is also possible to incorporate inorganic metals, such as copper or silver or even non-metals such as fluorine (F or Ca, particles previously mentioned), to enhance even further the antibacterial properties demonstrated by TiO_2_ coatings [29].

#### 3.2.4. Nanomaterials

Nanoparticles (NPs) are small particles with diameters between 1 and 100 nm from metals such as silver, gold, and other nanomaterials such as magnesium, zinc, or copper that display antimicrobial activity. Their antibacterial properties lead to research towards their incorporation in coatings for titanium implants [9,30].

The biocidal mechanisms shown by these nanoparticles, mainly the metallic ones, are diverse, which prevents bacteria in developing resistance against them [19]. Ag ions are released for a long period, expanding their antibacterial effect [31,32].

Massa et al. [33] incorporated Ag nanoparticles in a nanoporous silica coating through a sol–gel technique and observed a significant increase in both bactericidal and bacteriostatic properties of the titanium implant.

Silver nanoparticles (AgNPs) have shown a strong and wide antibacterial spectrum. Their exact mechanism against bacteria is still up for discussion, but the most accepted one so far is that AgNPs produce reactive oxygen species that inhibit the growth of bacteria, killing them in the process. For this reason, silveris one of the most used coating agents for titanium dental implants and other titanium medical devices [34]. However, some reports state that high concentrations of silver could be cytotoxic towards eukaryotic cells (e.g., fibroblasts and osteoblasts), which would reduce osseointegration properties of the implant [15]. Further studies are required to fully understand silver nanoparticles’ behavior when coated in titanium implants.

#### 3.2.5. Antibiotic Coatings

Nichol et al. [35] developed a single-layered sol–gel coating loaded with gentamicin on a titanium surface and tested it against *Staphylococcus* strains. Gentamicin is active against both Gram-negative and Gram-positive bacteria and is considered a broad-spectrum antibiotic.

Within 1 h, the Minimum Inhibition Concentration (MIC) was achieved, and after 24 h, all marked *Staphylococcus* variants were eliminated while 48 h later 99% of the gentamicin present in the coating was eluted [35].

These results were satisfactory to the author but do not represent the ideal coating for dental implants since antibiotic release was too fast for long-term prevention [35].

Zhang et al. [36] prepared titanium implants coated with vancomycin by using the electrospinning technique. Vancomycin was chosen due to its broad antimicrobial spectrum that covers both methicillin-resistant *S. epidermidis* as well as methicillin-resistant *S. aureus.*

The prepared coating showed an initial burst of vancomycin release on the first day (about 50.3%) followed by a slower and steadier release over the following 27 days (32.4%), making it a total release of approximately 528.2 μg of antibiotic from around 627.6 μg loaded in the coating (82.7%). No cytotoxicity to the cells was detected, and the antibacterial effect of Vancomycin was validated both in vitro and in vivo, showing promising results towards prevention of early implant-associated infections but still lacking long-term effects [36].

Lv et al. [37] also proposed an antibiotic-loaded coating to inhibit biofilm formation. They studied titanium substrates coated with a chitosan/alginate layer loaded with minocycline through layer-by-layer self-assembly. Minocycline is a broad-spectrum tetracycline antibiotic often used in conjunction with mechanical biofilm debridement in the treatment of periodontitis and peri-implantitis lesions.

This approach is extremely promising since the multilayered coating allows higher quantities of loaded antibiotics and a more controlled and over-time release of the substance.

The results obtained showed an initial burst of minocycline release in the first 24 h, which could fight the immediate colonization of bacteria. The antibiotic release stabilized during the first 7 days, and after that, the average concentration of minocycline on the fourteenth day was ~25.13 4.1 μg /mL. No bacterial cells with intact shape could be found on the titanium surface after 7 days [37].

In a recent systematic review by Souza et al. [38], all available references about antibiotic coated titanium surfaces were analyzed.

Out of those 33 articles, 11 used gentamicin, and 11 used vancomycin. Other antibiotics had three or fewer studies. *S.aureus* was the infection model of choice for 31 of the 33 studies [38].

Comparing the results obtained among all 33 articles, there was a big disparity from authors studying the same antibiotic. For example, in gentamicin-loaded coatings, bacterial reduction varied from ~5 up to ~99.9%. In vancomycin-loaded coatings, bacterial reduction ranged from ~45.3 up to ~99.2%. In three of the thirty-three studies, there were no reductions at all or even higher bacterial load in the tested group [38].

Bearing in mind the widespread and even contradictory range of results obtained and displayed in Table 1, as well as the scarce amount of data available, especially regarding human clinical data, there exists no consensual opinion regarding the best therapeutic approach for antibiotic-loaded coatings to prevent peri-implant infections [38]. There are also concerns towards toxicity and possible development of bacterial resistance, risks that should be avoided [15].

The fact that both gentamicin and vancomycin are not gold standards for the treatment of oral infection, since they act mostly on aerobic gram-negative bacilli, [38] result in the necessity to develop studies with antibiotics such as amoxicillin and metronidazole that would be more relevant for dental implants

Another important aspect to consider is that most of these studies were not conducted in the oral cavity or do not mimick its environmental conditions; thus, any conclusion regarding their behavior in dental titanium implants needs further studies [38].

#### 3.2.6. Silane

Silane is commonly used to induce surface modifications through a process designated as silanization, which allows the covalent attachment of various molecules (peptides, polymers, or proteins, for example) to the titanium surface [9,39].

Despite being used mainly as an anchor, some silanes have shown biological activities themselves. Buxadera-Palomero et al. [39], in a recent review, studied silane triethoxysilylpropyl succinic anhydride (TESPSA), which presents both osteoinductive and antibacterial activity. These authors compared uncoated titanium disks and TESPSA-coated disks, by using the silanization process, in vitro, using Streptococcus sanguinis and Lactobacillus salivaris cultures and even dental plaque collected from one volunteer. They accessed both cytotoxicity and antibacterial activity, and the results obtained demonstrate no signs of cytotoxicity and a significant reduction in bacterial adhesion even after 4 weeks of incubation when compared to uncoated disks. However, the results showed differences between the mono-species models and oral plaque, which proves the importance of using more than one biofilm model in these studies.

Ultimately, TESPSA-coated titanium presented great potential for dental applications after presenting a great antibacterial effect for a prolonged period.

#### 3.2.7. Nitride Coatings

Titanium nitride (TiN) is a material used to improve surface properties [9]. This material presents excellent chemical stability as well as resistance to corrosion and high temperatures while maintaining biocompatibility [9].

The antibacterial effect of a TiN and quaternized TiN (QTiN) coating surface on titanium was studied in vitro using *P. gingivalis* cultures [40]. The results obtained showed a significant reduction in bacterial coverage on TiN and QTiN coated surfaces after 4 h of culture. The uncoated group had 85.2% bacterial coverage while TiN-coated had only 24.22% and QTiN-coated only had 11.4% surface covered with bacteria after 4 h, while exhibiting good cell biocompatibility and promotion of osteoblast adhesion [40].

In another study, Ji et al. [41] found no antimicrobial effect in vitro by TiN against *P. gingivalis*; thus, further studies are required since the results are controversial.

#### 3.2.8. Chlorhexidine Coatings

Chlorhexidine has been used together with mechanical debridement to improve the effectiveness of treatment against peri-implantitis [9].

Lauritano et al. [42] studied the effectiveness of a silicone coating containing chlorhexidine against microbes inside and outside the implant-abutment junction (IAJ). They achieved the coated surface by immersion of the abutment in the polysiloxane solution for 10 min followed by centrifugation and heat treatment.

After 24 h incubation following contact with a microbial pool of *S.aureus*, *Escherichia coli*, *Pseudomonas aeruginosa*, and *Candida albicans*, the results showed no living microbes in the internal part of coated implants [42].

Considering the different approaches of coating the inside of the implant, preventing microbial growth in the IAJ, chlorhexidine also had promising results against the agents responsible for peri-implant infections in the short term [42].

### 3.3. New Perspectives on the Treatment of Peri-Implant Diseases

#### 3.3.1. Antisense Oligonucleotides (ASOs)

The antisense oligonucleotides (ASOs) are short fragments of a nucleic acid that can block a pre-defined target due to its complementarity. They were first described in 1978 by Zamecnik and Stephenson, who reported a blockage in viral replication and protein translation of a sarcoma virus RNA, after exposure to an antisense 13-nucleotide-long oligodeoxynucleotide in vitro [43]. Since this first generation of ASOs, several modifications have been made to overcome important limitations and enable clinical application such as incorporating 2′-O-methyl (2′-OMe) in the DNA backbone [44], connecting the DNA ribose ring by a methylene bridge between 2′-O and 4′-C atoms- locked nucleic acid (LNA) technology [45], or even using peptide nucleic acids (PNA) [46].

Antisense oligonucleotides can be used to interfere with essential biological processes of bacteria, which is helpful against bacterial infections. They can target many mRNA encoding essential genes, as well as functional domains of both 23S and 16S rRNA. Apart from targeting the essential mRNA and rRNA, ASOs can target non-essential genes related to biofilm formation, as many bacterial species form extracellular biofilms, making infections extremely challenging to eradicate. Some examples of biofilm-related genes are *motA* gene, encoding the element of the flagellar motor complex, the efaA gene, which plays an important role in the adhesion of bacteria to surfaces [46,47]. ASOs can also block two-component signal transduction systems, such as VicRK, that induce the gene expression for the synthesis of extracellular insoluble glucan, an extracellular matrix component of biofilms [48].

Biomaterial-based therapy has huge potential as biomaterial carriers can sustain slow-releasing drugs. Future development of new coating materials that can prevent the adhesion and subsequent biofilm formation of bacteria on dental implants, based on therapeutic antisense oligonucleotides, could be the key to fighting periodontitis. In 2020 Wu S. et al. developed a graphene oxide (GO)-based plasmid transformation system using electrostatic interacted GO-polyethyleneimine (PEI) complexes loaded with antisense vicR plasmid (GO-PEI-ASvicR). They showed that GO-PEI could efficiently deliver ASvicR plasmids into *S. mutans* cells with excellent transcripts of ASvicR, significantly reducing biofilm aggregation and exopolysaccharide (EPS) accumulation [48]. It is worth noting that graphene-based coating on titanium surfaces can be successfully obtained by electrodeposition as shown by Jankovic A (2015), making it a feasible option for innovative titanium coatings.

#### 3.3.2. Bacteriophages (Phages)

Bacteriophages (phages) are bacteria-infecting viruses that can detect specific receptors in bacteria, inject their genetic material, and exploit the host’s biochemical machinery to produce additional phage particles and enzymes, causing bacterial lysis. [49,50]. Shortly, the approach uses lytic pre-produced phages in a biodegradable drug delivery system to release and start their activity at the implant site [51]. Bacteriophages (phages) have emerged as a viable alternative to existing antimicrobial chemotherapy because of their ability to infect and kill specific bacterial strains while leaving the commensal microbiome intact [51]. The microbiota associated with a healthy (or commensal) state is more generalist, while disease-provoking microbiota is influenced by keystone microorganisms that have metabolic functions and an elevated virulence capacity that is mostly absent in healthy states [52]. As a result, we have reasons to believe that the use of bacteriophages (phages) would represent enhanced antimicrobial capacity, without compromising the microbiome associated with a healthy *periodontium*.

However, little evidence has been provided of the use of bacteriophages (phages) as therapy for dental implant-associated infections, limiting its application to the prevention and treatment of urinary catheters, respiratory ventilators, or orthopedic implant-associated infections [51,53,54].

#### 3.3.3. Antimicrobial Photodynamic Therapy (aPDT)

The main purpose of peri-implant disease treatment is to disinfect implant surfaces as well as supporting tissues, and non-surgical and surgical mechanical debridements with ultrasonic scalers or periodontal curettes are regarded as essential techniques for this purpose [55]. However, none of these approaches have proven to remove or at least inactivate these peri-implant infections due to the macroscopic and largely microscopic intricacy of the implant’s surface (rough and microporous) [56]. In addition to these methods, several studies have suggested that using adjuvant modalities, such as photodynamic therapy, can improve the treatment’s outcome [57,58].

Some research has looked into whether a synergistic combination of aPDT and coating materials (such as chitosan) can work as a synergistic antimicrobial agent against bacteria that trigger peri-implantitis, such as *S. aureus*, *E. coli*, and *P. aeruginosa* [56].

Antimicrobial photodynamic therapy (aPDT) gained popularity in the early twentieth century as a result of the work of Herman von Tappeiner’s team and is now used not only in medicine to treat certain tumors and skin diseases but also in dentistry to treat a variety of oral conditions such as peri-implantitis and peri-mucositis [58,59]. Photodynamic therapy (PDT) has been proved to be a successful treatment for peri-implantitis in the previous decade, owing to its ability to reach and penetrate the implant’s uneven surface [56]. The treatment consists of a reaction between an innocuous, non-invasive, and non-toxic photosensitizer (such as methylene blue or toluidine blue) combined with a low-energy light source in the presence of oxygen. For them to react, the light must have a precise wavelength that corresponds to the photosensitizer’s radiation absorption range, resulting in the creation of reactive oxygen species that are harmful to the bacterial cell and cause it to die. Gram-positive bacteria may be more vulnerable to this approach than Gram-negative bacteria due to the composition of their cell walls, making the photosensitizer more capable of invading those cells [60,61,62].

## 4. Discussion

### 4.1. Summary of Evidence

Our findings show a wide range of options and techniques to achieve an antimicrobial effect on the coating surface, as listed in Table 2. We cannot state which surface coating is the best based on the evidence since they all have their advantages and disadvantages. However, we believe that progress is being made towards improved dental implant solutions, but the ideal one, which promotes cell adhesion, biocompatibility, and antibacterial action overtime at a fair cost, is still a few years away.

Antibiotic coating of dental implants is highly preferred over other options according to our research, mostly because this option is closely linked to dental implant placement, as antibiotics are frequently recommended as a prophylactic medication for this procedure [36]. Even though there are several diverse protocols with various antibiotics, dosages, and administration times, the existing literature supports the benefits of prophylactic antibiotic therapy against implant failure due to immediate bacterial colonization [63]. The success rate of implant placement is higher when a prophylactic antibiotic is administrated to the patient, however, it only affects the early colonization of bacteria to the implant surface, not preventing biofilm establishment in the following days, weeks, or years. Not to mention, the actual amount of antibiotic that reaches the site of the implant is lower when compared to local delivery of the same antibiotic [35,36].

A surface coating with a controlled antibiotic delivery system demonstrates to be a great long-term solution to control and prevent biofilm formation. Specific agents released over time that target early colonizers without compromising the mechanical, physical, and chemical properties of the dental implant and showing non-cytotoxic effects to host tissues and cells would drastically decrease the occurrence of peri-implant infections [38].

Antibiotic coatings, on the other hand, have some drawbacks, the most significant of which are related to antibiotic resistance. As a result of their antibacterial and antimicrobial capabilities, we believe that alternative coating materials should be preferred as a viable alternative against biofilm colonization on titanium dental implants.

More than 700 species of bacteria populate the oral ecosystem [9,34] with *Actinobacteria*, *Bacteroidetes*, *Firmicutes,* and *Proteobacteria* being the most relevant for oral health [9]. Regarding dental biofilm, which may vary between individuals and even among different sites of the oral cavity, a core microbiome was proposed, and it included the following species: *Streptococcus*, *Veillonella*, *Granulicatella*, *Rothia*, *Actinomyces*, *Prevotella*, *Capnocytophaga*, *Porphyromonas*, and *Fusobacterium* [34,52].

The process of biofilm formation starts with salivary glycoproteins developing a conditioning film (also known as the acquired enamel pellicle) on the teeth surface, which allows initial bacterial adherence [34]. After that process, weak long-distance forces between charged molecules of the pellicle and the pioneer bacterial species will grant initial adhesion. These forces grow stronger via receptor pairs between adhesins in the bacteria’s surface and glycoprotein receptors in the acquired pellicle [34]. After the initial adhesion, biofilm development continues with cell aggregation until a stable microcolony is achieved. At last, due to multiple factors such as lack of nutrient or fluid dynamics, biofilm can disperse from the surface of the implant and migrate to other areas or tissues [64].

Medical devices, among others dental titanium implants, show a process of biofilm formation quite similar to natural teeth. A titanium surface, when present in the oral cavity, is immediately coated by plasma and saliva proteins. This will result in the formation of a protein layer that allows initial colonizers, such as various species of *Streptococcus*, to bind to it. Co-aggregation follows and interactions by different species induce biofilm accumulation. Finally, the extracellular matrix starts embedding microbial communities, and the biofilm is established [65].

Some materials such as silver have been used over the past few years as a coating material to reduce bacterial infections, but its use has been decreasing over time [15] due to concerns related to toxicity, which resulted in the switch to other solutions such as titanium dental implants with modified surfaces to improve osseointegration properties along with systemic administration of antibiotics, as we already discussed [15].

For the reasons and advantages stated, we propose that oligonucleotide probes should be considered as a feasible option for coating surface implants. We also suggest that future research should focus on determining whether the application of a combination of two or three different coating materials on the surface of dental implants can provide a synergic antimicrobial and antibacterial effect. Novel peri-implant therapies will result in a new and improved therapeutic approach and cannot be disregarded as an adjuvant approach of surface coatings. We anticipate that, by using these methods, we will be able to avoid the drawbacks of alternative coating materials (such as antibiotic resistance), prevent bacterial infections, and increase the success rate of dental implants.

### 4.2. Limitations

There is a lot of effort being placed into the discovery and applicability of established and new antimicrobial materials, but coating methods are complex and expensive.

Most of the mentioned coating possibilities are only applied in in vitro studies, and the ones that are employed in these conditions are not enough to establish clear conclusions. With so few in vivo trials, it is difficult to say when an efficient anti-fouling coating surface that would not drive up the price of a titanium dental implant will exist. We consider that more in vivo studies using relevant animal models and studies over longer periods are required for a better understanding of what is viable and what is not.

Many of the materials presented in this review were used as surface coatings for orthopedic implants. Despite being similar in most aspects, there are still quite a few relevant differences regarding orthopedic and dental implants. The oral cavity and oral microbiota are unique and distinct from the rest of the body, as we stated, and many of the results obtained require further investigation mimicking the environmental conditions of the oral cavity before they become possible solutions for dental implants.

## 5. Conclusions

All mentioned agents in this review have shown high levels of bacterial reduction when coated to titanium surfaces in vitro. However, the cost of fabrication, duration of the effect, and loss of osteoinductive properties appear to be the biggest obstacles faced to their broad appliance in titanium implants. Another major concern is the paucity of information on the bioactive surfaces’ long-term durability after implantation. The majority of the described methods have excellent outcomes, but only for the first 24 to 48 h following implantation. Some approaches, such as antibiotic-loaded coatings applied layer-by-layer, address this issue, but they are insufficient and require additional development to be a viable option.

Antibiotic coatings have shown the most promising results so far when taking into consideration the duration and antimicrobial effect combined with anti-fouling and antibiofilm properties. This coating material is the most popular; however, it faces a major drawback associated with the emergence of antibiotic resistance.

In conclusion, the evidence about ideal dental implants’ coating material is scarce and further studies are required before presenting more consolidated conclusions. More high-quality randomized clinical trials (RCTs) with longer follow-up periods, more precise criteria, and better-described coating protocols oriented to oral-biofilm-induced diseases are needed to establish a standardized guideline for this therapy’s application. It is also critical to conduct more research comparing the application of titanium dental implant’s coatings adjuvant to other complementary treatments for peri-implant diseases, such as antimicrobial photodynamic therapy, in order to establish which ones offer the most benefits. Furthermore, we highlight that antimicrobial and antibiofilm coatings applied to the surface of dental implants must not harm the microbiota associated with a healthy *periodontium*.

## Figures and Tables

**Table 1:** Summary of results obtained with different antibiotics.

Antibiotic	Model	Efficiency	Reference
Gentamycin	*Staphylococcus* variants	~99% (24 h)	[36]
	*S. aureus*	From ~5 to ~99%	[39]
Vancomycin	*S. epidermidis and S. aureus*	Significant reduction (non-specified)	[37]
	*S. aureus*	From ~45.3 to ~99.2%	[39]
Minocycline	*S. aureus*	~99% (7 days) and ~80% (14 days)	[38]
	*S. aureus*	Non-reported	[39]

**Table 2:** Synthesis of the gathered evidence.

Coating Surface	Mechanism of Action	Major Upside(s)	Major Downside(s)
Polymer Coatings	Bacteriostatic (mainly)/Bactericidal	Great anti-biofouling and osseointegration properties when paired with cell-adhesive sequences; great bacteriostatic results in vitro	Risk of polymer degradation; require pairing with cell adhesive sequences
Antimicrobial Peptides	Bactericidal	Broad spectrum; low cytotoxicity; low propensity to develop antibiotic resistance	Complex structure; high cost of fabrication
Ion-implanted Surfaces	Bactericidal	Flexibility; can be paired with other coatings to promote both osseointegration and anti-biofouling properties	Difficulty to achieve a long-term antimicrobial effect
Photoactivatable Bioactive Titanium	Bactericidal	Cheap; stable; biocompatibility	Inability to photoactivate once the implantation occurs
Nanomaterials	Bacteriostatic (mainly)/Bactericidal	Longer antimicrobial effect	Efficiency is controversial; some studies report cytotoxicity
Totarol	Bacteriostatic (mainly)/Bactericidal	Efficient and long antimicrobial effect	Biodegradable substance
Antibiotic Coatings	Bactericidal	Cheap; good efficiency against targeted bacteria	Development of bacterial resistance; difficulty to achieve long-term release; toxicity
Chlorhexidine Coatings	Bactericidal	Great results in vitro regarding biofilm reduction	Absorption by the titanium surface
Biosurfactants	Bacteriostatic	Some bactericidal effects, increasing effectiveness	Scarce studies
Nitride Coatings	Bactericidal	Promotion of osteoblast adhesion while maintaining the antimicrobial effect	Controversial results against bacteria present in the oral cavity
Silane	Bactericidal	Combination of antibacterial effect and osteoinductive properties	Require further studies with different biofilm models
Antisense Oligonucleotides (ASOs)	Bacteriostatic	Can be used to interfere with essential biological processes of bacteria	The complex design of the probes to avoid low affinity to the target
Bacteriophages	Bactericidal	Having the ability to infect and kill specific bacterial strains while leaving the commensal microbiome intact	Little evidence has been provided in dental implant-associated infections
